# Potential role of *FKBP5* single‐nucleotide polymorphisms in functional seizures

**DOI:** 10.1002/epi4.12716

**Published:** 2023-03-21

**Authors:** Ali A. Asadi‐Pooya, Leila Simani, Marjan Asadollahi, Fatemeh Sadat Rashidi, Ehsan Ahmadipour, Afagh Alavi, Mehrdad Roozbeh, Nayyereh Akbari, Negar Firouzabadi

**Affiliations:** ^1^ Epilepsy Research Center Shiraz University of Medical Sciences Shiraz Iran; ^2^ Jefferson Comprehensive Epilepsy Center, Department of Neurology Thomas Jefferson University Philadelphia Pennsylvania USA; ^3^ Brain Mapping Research Center Shahid Beheshti University of Medical Sciences Tehran Iran; ^4^ Department of Pharmaceutical Sciences, College of Pharmacy University of Kentucky Lexington Kentucky USA; ^5^ Department of Epilepsy, Loghman Hakim Hospital Shahid Beheshti University of Medical Sciences Tehran Iran; ^6^ Neuroscience Research Center Shahid Beheshti University of Medical Sciences Tehran Iran; ^7^ Genetics Research Center The University of Social Welfare and Rehabilitation Sciences Tehran Iran; ^8^ Department of Pharmacology & Toxicology School of Pharmacy, Shiraz University of Medical Sciences Shiraz Iran

**Keywords:** dissociative, genetic, nonepileptic, psychogenic, seizure

## Abstract

**Objective:**

We investigated the associations between *FKBP5* single‐nucleotide polymorphisms (SNPs) and functional seizures (FS).

**Methods:**

Seventy patients with FS, 140 with major depressive disorder (MDD), and 140 healthy controls were studied. Their DNAs were analyzed for the rs1360780 in the 3′ region and rs9470080 in the 5′ region of the *FKBP5*. Childhood trauma questionnaire and hospital anxiety and depression scale were used.

**Results:**

Patients with FS and those with MDD had less GG and more AA genotypes in both rs9470080 and rs1360780 SNPs compared with those in healthy controls. Similar results were observed for allelic frequencies. There were no significant differences between FS and MDD groups in terms of genotype and allelic frequencies for both SNPs. The results of multinomial logistic regression analysis showed that *FKBP5* polymorphisms were not associated with the diagnosis.

**Significance:**

Patients with FS and those with MDD had significantly different genotypes in both rs9470080 and rs1360780 SNPs compared with those in healthy controls. However, it seems that *FKBP5* polymorphisms were not associated with FS in the absence of depression. Further genetic investigations of patients with FS may increase our understanding of the neurobiological underpinnings of this condition, but such studies should be large enough and very well designed; they should include a comparison group with depression in addition to a healthy control group.

**Key points**

- Patients with FS and those with MDD had significantly different genotypes in both rs9470080 and rs1360780 SNPs compared with those in healthy controls.
- However, it seems that *FKBP5* polymorphisms were not associated with FS in the absence of depression.
- We should include a comparison group with depression, in addition to a healthy control group, in any future genetic study of patients with FS.

## 1 INTRODUCTION

Functional seizures (FS) are commonly encountered at epilepsy centers and neurology clinics.^1^ These include sudden changes in motor functions, responsiveness, or behavior that may resemble epileptic seizures, but are not accompanied by ictal epileptic changes in electroencephalography (EEG); they are commonly associated with psychological problems.^2^, ^3^, ^4^ Knowledge on the biological reasons of FS is still scarce; but, there is growing evidence about abnormal structural and functional brain connectivity in patients with FS.^5^ Patients with FS commonly have psychiatric comorbidities [e.g., depression or posttraumatic stress disorder (PTSD)]^6^ and genetic factors play a significant role in the pathophysiology of these psychiatric disorders, in general.^7^, ^8^

FK506‐binding protein 51 or *FKBP5* is a co‐chaperone of hsp90, which regulates glucocorticoid receptor (GR) sensitivity.^9^ *FKBP5* is induced by cortisol and acts within a negative feedback loop to promote the transcription of stress‐responsive target genes, leading to downstream release of adrenocorticotropic hormone (ACTH) and cortisol.^10^, ^11^ *FKBP5* is located on chromosome 6p21 and contains a number of single‐nucleotide polymorphisms (SNPs) that are associated with differential ability for *FKBP5* to be induced by cortisol and bind to the GR.^12^, ^13^, ^14^ *FKBP5* SNPs have been associated with an increased risk of various psychiatric disorders.^15^, ^16^, ^17^, ^18^, ^19^ Furthermore, while *FKBP5* SNPs are not linked with the risk of experiencing childhood trauma, they may contribute to its sequelae. Interactions between *FKBP5* gene and early‐life traumatic experiences (e.g., childhood sexual trauma, which is also a significant risk factor for FS^5^) may increase the likelihood of stress‐related disorders later in the life.^18^ The interactions between childhood sexual trauma and *FKBP5* SNPs have been related to the altered glucocorticoid (cortisol) levels and a heightened threat‐related amygdala reactivity.^20^, ^21^

In the current endeavor, we aimed to investigate whether there are associations between two common *FKBP5* polymorphisms (rs1360780 in the 3′ region and rs9470080 in the 5′ region of the *FKBP5*) and FS in a case–control study. We hypothesized that the tested *FKBP5* polymorphisms have significant associations with FS, independent from their comorbid depression.

## 2 METHODS

### 2.1 Participants and study design

We conducted a cross‐sectional case–control study on 70 people with FS (who were admitted at Loghman Hakim Hospital, Tehran, Iran, from 2020 until 2021 or at Shiraz Comprehensive Epilepsy Center at Shiraz University of Medical Sciences, Shiraz, Iran from 2020 until 2021) and 140 persons with major depressive disorder (MDD) (confirmed via clinical assessment and diagnosed according to the DSM‐V criteria by a psychiatrist, who were referred to Loghman Hakim Hospital outpatient psychiatry clinic in Tehran, Iran, from January 2020 through September 2020), as well as 140 healthy controls (HC) from the same cities (people with no history of seizures and no history of psychiatric disorders—self report). Since depression has significant associations with FS and also since *FKBP5* SNPs have been associated with an increased risk of depression, we included two control groups (MDD and HC) to investigate the confounding effects of depression on the results. During the study, the COVID‐19 pandemic created significant difficulty in recruiting patients with FS in video‐EEG monitoring units; therefore, we included half as many FS as the other two groups. We included adults 18 years to 50 years of age. The study was approved by the Research Ethics Committee of Neuroscience Research Center, Shahid Beheshti University of Medical Sciences, Tehran, Iran (IR.SBMU.PHNS.REC.1399.007). All the participants gave their written informed consent before participating in the study.

Patients with a documented diagnosis of FS (based on the levels of diagnostic certainty that were previously published^22^), determined by clinical assessment and video‐EEG monitoring with ictal recording, were included. The epileptologists interviewed all the patients. We classified FS into two distinct semiological classes (based on the most common type of the habitual seizures of the patients): (a) generalized motor: events were mainly characterized by tonic, clonic, or dystonic generalized movements, tremors, rigor‐like movements, whole‐body rigidity, pelvic thrusting, pedaling, or side to side head movements; (b) akinetic: events were mainly characterized by unresponsiveness and the absence of movement.^23^

Age, sex, age at functional seizure onset, risk factors potentially predisposing to FS [e.g., a history of physical abuse, a history of sexual abuse (rape), a history of family dysfunction (i.e., divorce, single parent, significant family disputes, etc.), a history of academic failure (school dropout or repeated grades), and family history of any seizures], and video‐EEG recording of all patients with FS were registered in the database. Other clinical and demographic variables (i.e., education, marital status, and parental consanguinity) were extracted from the medical records. The Persian validated Hospital Anxiety and Depression Scale (HADS)^24^ and Childhood Trauma Questionnaire (CTQ) scale^25^ were used in all the participants (all three groups). The risk factors potentially predisposing to FS were assessed both via clinician interview and CTQ scale. The clinical diagnosis of depression in the MDD group was confirmed via clinical assessment and diagnosed according to the DSM‐V criteria, but in order to compare the groups we applied HADS in all three groups of the study.

### 2.2 Genetic analysis

Given that a variety of *FKBP5* SNPs have been investigated in different studies of patients with psychiatric problems, we only selected those SNPs that were reported to have higher risks for depressive disorders, PTSD, or suicidal behavior^17^, ^18^ in meta‐analysis studies; as a result, only two SNPs were selected: rs1360780 in the 3′ region and rs9470080 in the 5′ region of *FKBP5* gene. DNA were extracted from the venous blood collected in EDTA tubes using the salting‐out method. The extracted DNA was normalized to a concentration of 150 ng/ul. The DNA fragments containing the rs9470080 and rs1360780 SNPs were amplified by polymerase chain reaction (PCR) using Taq DNA polymerase 2× Master Mix (Ampliqon—Denmark, www.ampliqon.com) procedure and with specific primers (Table S1) in a total volume of 25 μL. Primers were designed using Primer‐BLAST (https://www.ncbi.nlm.nih.gov/tools/primer‐blast/). All PCR reactions were done on a ABi Thermocycler under an initial denaturation step for 5 min at 95°C, 35 cycles of denaturation at 95°C for 30 s, annealing at 60°C for 30 s, and extension at 72°C for 30 s, followed by a final extension step of 72°C for 5 min. PCR products were separated on a 1% agarose gel to distinguish the allele 288 bp for rs9470080 SNP and the allele 304 bp for rs1360780 SNP, then Sanger sequenced using the Sanger method (Big Dye kit Prism 3130 sequencer; Applied Biosystems, Foster City, CA, USA).

### 2.3 Statistical analyses

Data were analyzed using SPSS 21.0 for Windows (SPSS Inc., Chicago). Distribution of all continuous variables was tested for normality with Kolmogorov–Smirnov test. Chi‐square, independent *t*‐test, one‐way ANOVA (LSD post‐test), and Kruskal–Wallis (Dunnett post‐test) were used to compare quantitative and categorical variables. Differences among FS, MDD, and healthy control groups for HADS and CTQ scale were measured using ANOVA with post hoc last significant difference (LSD) test. The genotype distributions of chosen SNPs were tested for deviation from Hardy–Weinberg equilibrium (HWE) in all groups. Genotype and allelic distribution between groups was analyzed by Chi Square (*χ* ^2^) test. Furthermore, a multinomial logistic regression analysis was performed to predict the relationship between the diagnosis, genotype, CTQ, and HADS. Also, a linear model regression analysis was applied in FS group to predict disease characteristics (seizure frequency and disease duration) ~ genotype + HADS + CTQ scales. The Benjamin–Hochberg procedure statistical control for multiple comparisons was used to combat family‐wise error. Continuous variables are demonstrated as mean ± standard deviation (SD). A *p* value <0.05 was considered statistically significant. The odds ratios (ORs) and 95% confidence intervals (CIs) were calculated.

## 3 RESULTS

### 3.1 Participants characteristics

As summarized in Table 1, no significant differences were found in the main demographic variables (i.e., sex and age) between the groups. However, the groups differed significantly with respect to some characteristics (i.e., education level, marital status, and parental consanguinity). Among 70 patients with FS, 49 persons (70%) had generalized motor events and 21 people (30%) had akinetic events. The mean FS frequency was 17.67 ± 18.25 episodes per month and the disease duration before the final diagnosis was 7.14 ± 7.39 years. Following post hoc Dennett's test, significant differences were observed in CTQ scale and HADS scores: patients with MDD had higher scores on both scales compared with those with FS and heathy controls and patients with FS had higher scores compared with healthy controls.

**TABLE 1:** Demographic and clinical characteristics of the participants (% are in the parenthesis).

Variable	FS: *N* = 70	MDD: *N* = 140	HCs: *N* = 140	*p*‐value
Male	23 (32.9)	46 (32.9)	60 (42.9)	0.154
Female	47 (67.1)	94 (67.1)	80 (57.1)	
Mean age (SD) (years)	32.45 (10.11)	32.0 (8.50)	33.33 (6.92)	0.143
Elementary school	27 (38.6)	47 (33.6)	24 (17.1)	0.002
High school diplom	34 (48.6)	59 (42.1)	80 (57.1)	
College	9 (12.9)	34(24.3)	36 (25.7)	
Single	24 (34.3)	74 (52.9)	59 (42.4)	0.009
Married	43 (61.4)	52 (37.1)	73 (52.5)	
Separated	3 (4.3)	14 (10)	7 (5)	
Disease duration (SD) (years)	7.14 (7.39)	6.56 (6.39)	–	0.576
Family history of seizures	17 (24.3)	34 (24.3)	–	0.978
Parental consanguinity	23 (32.9)	32 (22.9)	14 (14.9)	0.014
Physical abuse	14 (20)	71 (50.7)	7 (5)	0.001
Sexual abuse	13 (18.6)	19 (13.6)	–	0.217
Family dysfunction	21 (30)	105 (76.1)	11 (7.9)	0.001
HADS score (SD)	23.10 (8.56)	26.10 (5.9)	10.52 (6.49)	0.001^a^, 0.01^b^
CTQ score (SD)	62.48 (17.05)	69.17 (14.91)	45.61 (10.03)	0.001^a^, 0.01^b^

*Note*: Independent sample t‐test (disease duration), chi‐square, and ANOVA with post hoc last significant difference (LSD) test were used to explore the differences among the FS, MDD, and healthy controls (quantitative and categorical data). In addition, Kruskall–Wallis with post hoc Dennett's test was used for nonparametric variables.

Abbreviations: CTQ, Childhood Trauma Questionnaire; FS, functional seizures; HADS, Hospital Anxiety and Depression Scale; HC, healthy controls; MDD, major depressive disorder; SD, standard deviation.

^a^ HC vs. FS, MDD.

^b^ FS vs. MDD.

### 3.2 Genetic analysis

Controls and patients were in Hardy–Weinberg equilibrium for rs9470080 (*χ* ^2^ = 3.37, *p* = 0.066) and rs1360780 (*χ* ^2^ = 0.25, *p* = 0.61). Tables 2 and 3 demonstrate the genotypic distributions and allelic frequencies of the rs9470080 and rs1360780 G>A. As the data show, patients with FS and those with MDD had less GG and more AA genotypes in both rs9470080 and rs1360780 SNPs compared with those in healthy controls (Table 2). Similar results were observed between allelic frequencies of the healthy controls and patients for both SNPs. Patients with FS and MDD had more A allelic frequencies and less G allelic frequencies (Table 3). There were no significant differences between FS and MDD groups in terms of genotype and allelic frequencies for both SNPs (*p* > 0.05 for all comparisons). The results of multinomial logistic regression analysis showed that *FKBP5* polymorphisms were not associated with the diagnosis (Table 4). In a separate analysis, there was no statistically significant association between both genotypes, HADS score, seizure frequency, and disease duration in multiple linear regression model of FS group (Table 5). Finally, there were no statistically significant associations between different genotypes and alleles of rs9470080 and rs1360780 and the semiological classes of FS (*p* = 0.07 and 0.05, respectively).

**TABLE 2:** Genotype frequencies (*χ* ^2^) of the investigated SNPs among the studied groups.

Polymorphism and its genotype frequency	rs9470080				rs1360780			
The analyzed groups	AA (*n*) (%)	AG (*n*) (%)	GG (*n*) (%)	*p*‐ value	AA (*n*) (%)	AG (*n*) (%)	GG (*n*) (%)	*p*‐ value
Healthy controls	6 4.3	53 37.9	81 57.9	0.006	8 5.7	53 37.9	79 56.4	0.01
FS	9 12.9	34 48.6	27 38.6		10 14.3	29 41.4	31 44.3	
MDD	9 6.4	74 52.5	57 41.1		13 9.2	71 50.4	56 40.4	

*Note*: The *p*‐values represent the differences between the groups.

Abbreviations: FS, Functional seizures; MDD, Major depressive disorder; SNP, Single nucleotide polymorphism.

**TABLE 3:** Allelic frequencies (*χ* ^2^) of the investigated SNPs among the studied groups.

Polymorphism and its allele frequency	rs9470080			rs1360780		
Analyzed groups	A (*n*) (%)	G (*n*) (%)	*p*‐ value	A (*n*) (%)	G (*n*) (%)	*p*‐value
Controls	65 22.4	215 77.6	0.005	69 24.6	211 75.4	0.02
FS	52 37.6	88 62.4		49 35	91 65	
MDD	92 32.6	188 67.4		97 34.4	183 65.6	

*Note*: The *p*‐values represent the differences between the groups.

Abbreviations: FS, functional seizures; MDD, major depressive disorder; SNP, Single‐nucleotide polymorphism.

**TABLE 4:** Results of the multinomial logistic regression analysis.

Group^a^	Odds ratio (OR)	95% CI	*p*‐value
FS			
rs1360780 (GG)	0.605	0.027–13.469	0.995
rs1360780 (AG)	0.332	0.017–6.486	0.995
rs1360780 (AA)^b^	–	–	–
rs9470080 (GG)	0.385	0.013–11.028	0.995
rs9470080 (AG)	1.011	0.042–24.087	0.995
rs9470080 (AA)^b^	–	–	–
CTQ score	1.055	1.024–1.087	0.005
HADS score	1.200	1.133–1.271	0.005
MDD			
rs1360780 (GG)	0.117	0.007–2.097	0.446
rs1360780 (AG)	0.206	0.013–3.284	0.446
rs1360780 (AA)^b^	–	–	–
rs9470080 (GG)	3.450	0.143–83.184	0.446
rs9470080 (AG)	3.462	0.166–72.361	0.446
rs9470080 (AA)^b^	–	–	–
CTQ score	1.076	1.045–1.108	0.005
HADS score	1.262	1.190–1.338	0.005

*Note*: *p* value adjusted with Benjamin–Hochberg.

Abbreviations: CTQ, Childhood Trauma Questionnaire; FS, functional seizures; HADS, Hospital Anxiety and Depression Scale; MDD, major depressive disorder.

^a^ The reference category is: Healthy.

^b^ This parameter is set to zero because it is redundant.

**TABLE 5:** Association between seizure frequency, disease duration, genotypes, and HADS in multiple linear regression model of FS group.

Variable		Crude		Adjusted	
	Seizure frequency	β coefficient	*p*‐value	β coefficient	*p*‐value
HADS		−0.238	0.340	−0.252	0.306
rs1360780					
GG	17.25 ± 17.55	−0.807	0.797	−15.99	0.50
AG	19.65 ± 20.70				
AA (reference)	13.50 ± 12.57				
rs9470080					
GG	14.81 ± 16.08	1.621	0.622	17.22	0.49
AG	20.82 ± 20.81				
AA (reference)	14.66 ± 12.74				

	Disease duration	β coefficient	*p*‐value	β coefficient	*p*‐value
HADS		0.038	0.708	0.033	0.743
Rs1360780					
GG	7.96 ± 8.03	−1.873	0.137	0.802	0.820
AG	7.62 ± 7.52				
AA (reference)	3.20 ± 2.69				
Rs9470080					
GG	8.51 ± 8.19	−2.245	0.089	−2.245	0.093
AG	7.02 ± 7.36				
AA (reference)	3.44 ± 2.74				

Abbreviations: FS, functional seizures; HADS, Hospital Anxiety and Depression Scale.

## 4 DISCUSSION

In the present study, we investigated the genotype distributions of two common *FKBP5* polymorphisms (rs9470080 and rs1360780) in Iranian patients with FS. Patients with FS and those with MDD had significantly different genotype and allelic frequencies for both tested SNPs compared with those in healthy controls. However, there were no significant differences between FS and MDD groups in terms of genotype and allelic frequencies for both SNPs. Forty‐eight patients (68.6%) from the FS group had high scores on HADS and all patients with MDD reported significant levels of depressive symptoms. Our results showed that *FKBP5* polymorphisms were not associated with FS in the absence of depression. The results of our study highlight the need to include a comparison group with depression (and probably, PTSD or anxiety), in addition to a healthy control group, in any future genetic study of patients with FS.

A recent study generated whole‐exome sequencing and whole‐genome genotyping data to identify rare, pathogenic (P) or likely pathogenic (LP) variants in 102 patients with FS and 448 individuals with epilepsy; they observe that six (5.9%) individuals with FS carried P/LP variants.^26^ Considering this study and also our observations, it seems that further genetic investigations of patients with FS may increase our understanding of the neurobiological underpinnings of this condition and may lead to new horizons in the field.

In addition to their associations with various psychopathologies, *FKBP5* genotypes are also associated with alterations in the brain function and structure, especially in the brain regions that are associated with emotional processing, learning, memory, and inhibition (i.e., amygdala and hippocampus).^21^, ^27^, ^28^, ^29^ In one study, in the rodent brain, basal *FKBP5* expression was the highest in the hippocampus, but strong induction of *FKBP5* was observed in the amygdala and the paraventricular nucleus of the hypothalamus after a stress or glucocorticoid challenge.^30^ Furthermore, widespread structural changes in the subcortical and cortical emotion‐processing brain areas have been related to *FKBP5* and childhood abuse.^31^ Therefore, considering and investigating *FKBP5* polymorphisms in patients with FS in large and well‐designed studies may be revealing.

### 4.1 Limitations

This study has some significant limitations. The most important shortcoming of our study was its relatively small sample size; hence, the findings should be interpreted with caution. Also, we did not clinically assess a comorbid diagnosis of MDD in those with FS (we used HADS). In addition, we did not consider other potentially important confounding variables such as PTSD. Furthermore, this study was based on a single country, which may limit its generalizability to other populations. Finally, our analyses tested only two SNPs within *FKBP5*; other alterations were not investigated.

## 5 CONCLUSION

Patients with FS and those with MDD had significantly different genotypes in both rs9470080 and rs1360780 SNPs compared with those in healthy controls. However, it seems that *FKBP5* polymorphisms were not associated with FS in the absence of depression. Further genetic investigations of patients with FS may increase our understanding of the neurobiological underpinnings of this condition, but such studies should be large enough and very well designed; they should include a comparison group with depression (and probably, PTSD or anxiety) in addition to a healthy control group.

## AUTHOR CONTRIBUTIONS

Ali A. Asadi‐Pooya, M.D.: Study conceptualization and design, data collection, and manuscript preparation. Leila Simani Ph.D.: Data collection, statistical analyses, and manuscript preparation. Others: Data collection and manuscript preparation.

## FUNDING INFORMATION

This research was partially supported by grants from Shiraz University of Medical Sciences and the Brain Mapping Research Center of Iran. The funding sources was not involved in study design; in the collection, analysis and interpretation of data; in the writing of the report; and in the decision to submit the article for publication.

## CONFLICT OF INTEREST STATEMENT

Ali A. Asadi‐Pooya: Honoraria from Cobel Daruo, Tekaje, and RaymandRad; Royalty: Oxford University Press (Book publication); Grant from the National Institute for Medical Research Development. Others: no conflict of interest.

## ETHICAL APPROVAL

The study was approved by the Research Ethics Committee of Neuroscience Research Center, Shahid Beheshti University of Medical Sciences, Tehran, Iran (IR.SBMU.PHNS.REC.1399.007). All the participants gave their written informed consent before participating in the study. Consent for publication is not applicable to this study.

## Supporting information


Table S1.

## Data Availability

The data used in this study are confidential and will not be shared as per regulations of Shiraz University of Medical Sciences.
