# Supplementary material for: Potential role of FKBP5 single‐nucleotide polymorphisms in functional seizures
Source: Epilepsia Open. 2023 Mar 21;8(2):479–86. doi: 10.1002/epi4.12716 (PMC10235573; doi:10.1002/epi4.12716)
Supplement: Supplementary file 1 — Table S1. [file EPI4-8-479-s001.docx]

| **Supplementary Table 1.** Primers used for polymerase chain reaction (PCR) and sequencing of the candidate single nucleotide polymorphisms (SNPs). | |
| --- | --- |
| **SNP** | **Primers sequence (5'→3')** |
| rs9470080 | F-CTCACAGAGATCATAAAACAGTGG |
|  | R-CAAACTTTCCAGATGAACAGTACC |
| rs1360780 | F-GAATCTGAGAAAGGTTAAGTGG |
|  | R-CTTATTCTATAGCTGCAAGTCC |
